# Oral Serum-Derived Bovine Immunoglobulin/Protein Isolate Has Immunomodulatory Effects on the Colon of Mice that Spontaneously Develop Colitis

**DOI:** 10.1371/journal.pone.0154823

**Published:** 2016-05-03

**Authors:** Anna Pérez-Bosque, Lluïsa Miró, Mònica Maijó, Javier Polo, Joy M. Campbell, Louis Russell, Joe D. Crenshaw, Eric Weaver, Miquel Moretó

**Affiliations:** 1 Departament de Bioquímica i Fisiologia, Facultat de Farmàcia i Ciències de l’Alimentació and Institut de Nutrició i Seguretat Alimentària, Universitat de Barcelona (UB), Barcelona, Spain; 2 APC Europe S.A., Granollers, Spain; 3 APC Inc, Ankeny, IA, United States of America; 4 EnteraHealth, Cary, NC, United States of America; Jawaharlal Nehru University, INDIA

## Abstract

Dietary immunoglobulin concentrates prepared from animal plasma can modulate the immune response of gut-associated lymphoid tissue (GALT). Previous studies have revealed that supplementation with serum-derived bovine immunoglobulin/protein isolate (SBI) ameliorates colonic barrier alterations in the mdr1a-/- genetic mouse model of IBD. Here, we examine the effects of SBI on mucosal inflammation in mdr1a-/- mice that spontaneously develop colitis. Wild type (WT) mice and mice lacking the mdr1a gene (KO) were fed diets supplemented with either SBI (2% w/w) or milk proteins (Control diet), from day 21 (weaning) until day 56. Leucocytes in mesenteric lymph nodes (MLN) and in *lamina propria* were determined, as was mucosal cytokine production. Neutrophil recruitment and activation in MLN and *lamina propria* of KO mice were increased, but were significantly reduced in both by SBI supplementation (*p* < 0.05). The increased neutrophil recruitment and activation observed in KO mice correlated with increased colon oxidative stress (*p* < 0.05) and SBI supplementation reduced this variable (*p* < 0.05). The Tact/Treg lymphocyte ratios in MLN and *lamina propria* were also increased in KO animals, but SBI prevented these changes (both *p* < 0.05). In the colon of KO mice, there was an increased production of mucosal pro-inflammatory cytokines such as IL-2 (2-fold), IL-6 (26-fold) and IL-17 (19-fold), and of chemokines MIP-1β (4.5-fold) and MCP-1 (7.2-fold). These effects were significantly prevented by SBI (*p* < 0.05). SBI also significantly increased TGF-β secretion in the colon mucosa, suggesting a role of this anti-inflammatory cytokine in the modulation of GALT and the reduction of the severity of the inflammatory response during the onset of colitis.

## Introduction

Inflammatory bowel diseases (IBD) are characterized by diffuse chronic intestinal inflammation, typically involving neutrophil infiltration, the production of inflammatory mediators, and alterations in the colon barrier that are usually life-long in nature [1]. The etiopathogenesis of IBD has not been clearly elucidated, but it involves a complex interplay of genetic, environmental, microbial, and immune factors, which alter the barrier properties of the mucous and epithelial layers. This allows luminal toxins and antigens to penetrate the mucosa and challenge the gut-associated lymphoid tissue (GALT). Dysregulation of gut immunity causes an overproduction of pro-inflammatory cytokines and trafficking of effector leukocytes into the intestinal mucosa, thus leading to an uncontrolled intestinal inflammation [2].

The treatment of IBD is mainly pharmacological and involves anti-inflammatory drugs and agents that reduce the symptoms associated with IBD [3]. These therapies ameliorate IBD, but their use for long periods of time may result in adverse side effects, including immune suppression [4]. For these reasons, nutrition emerges as an alternative to drug therapies, and food choice has become a promising tool for the treatment and prevention of IBD [5]. In this respect, dietary supplementation with specific macro- and micronutrients such as omega-3 fatty acids [6], prebiotics and probiotics [7] or polyphenolic compounds [8] has been shown to have some effectiveness. Milk-derived supplements have also been evaluated for the treatment of inflammatory syndromes. For example, bovine colostrum improved clinical symptoms of colorectal inflammation in a well-established mouse model of DSS-induced colitis [9], and lactoferrin [10] and glycomacropeptide from bovine milk [11] demonstrated anti-inflammatory properties in rodent models of colitis. Animal plasma-derived proteins also have anti-inflammatory effects and are candidates for use in the management of IBD. Results from our laboratory show that dietary supplementation with porcine spray-dried plasma proteins, either full plasma (spray-dried plasma, SDP) or an immunoglobulin-rich concentrate (IC), can regulate GALT-mediated immune responses in models of acute intestinal inflammation. In the small intestine, both SDP and IC reduce the activation of Th lymphocytes [12], prevent the release of pro-inflammatory cytokines [13] and restore impaired barrier function [14], in a rat model of intestinal inflammation induced by the *S*. *aureus* enterotoxin B.

Another supplement that has been extensively studied in animal models is serum-derived bovine immunoglobulin/protein isolate (SBI), which contains over 90% protein, more than 50% of which consists of immunoglobulins, mainly IgG. SBI reduces inflammatory markers and tissue damage in mice models of colitis [15] and mucositis [16], and it has been reported to be effective in the management of enteropathy associated with diarrhea-predominant IBS and HIV infection [17]. Clinical trials in which SBI was administered to patients with irritable bowel syndrome obtained some improvements in symptoms, consistent with a limiting effect of SBI on cytokine production in the inflamed intestinal mucosa [18], through a mechanism involving bacterial antigen binding in the intestinal lumen [19].

In this study, the effects of SBI administration on colitis were assessed in mice lacking the mdr1 gene. This gene encodes P-glycoprotein 170 (P-gp), a membrane transporter that actively pumps toxins and xenobiotics back to the lumen and which is highly expressed in the gastrointestinal tract [20]. The mdr1a-/- mouse is a good model of colitis because it shows barrier alterations and inflammation with a location and characteristics similar to human IBD [21], and in particular to Crohn’s disease (CD). Moreover, in inflamed mdr1a-/- mice, changes in the expression of genes coding for pro-inflammatory markers and detoxification enzymes are similar to those observed in human IBD, further supporting the suitability of this model for pre-clinical studies [22].

Using this model, dietary SBI has been shown to attenuate barrier alterations in the colon, reduce the expression of pro-inflammatory cytokines such as TNF-α and IFN-γ, and the expression of iNOS [15], all of which are involved in the regulation of mucosal integrity. In view of these effects, it was decided to expand the study of the immunomodulatory functions of SBI in mdr1a-/- mice by analyzing the profile of T-lymphocyte populations and the expression of other pro-inflammatory mediators and anti-inflammatory cytokines in mesenteric lymph nodes (MLN) and the *lamina propria* of the colon.

## Material and Methods

### Animals and diets

FVB wild type mice (WT) and mdr1a-/- mice (FVB.129P2-Abcb1atm 1BorN7; KO) were purchased from Taconic (Germantown, NY). A stable colony was obtained and maintained in the specific pathogen free (SPF) area of the Animal Experimentation Service of the Barcelona Science Park (BSP), University of Barcelona. Animals were kept under stable temperature and humidity conditions, with a 12 h:12 h light/dark cycle. Mice were housed in polycarbonate microisolator cages contained in individual ventilated racks under barrier conditions. All protocols used in this study were approved by the Ethics Committee for Animal Experimentation of the BSP (Permit number: P03-R1-08). In accordance with our institution’s guidelines, the number of animals used in our study was kept as low as possible, usually 7–8 mice per group, and treated under blind conditions. Special care was taken to prevent infection with environmental *Helicobacter* bacteria and animals were checked to ensure they were *Helicobacter* free using sentinel mice.

Serum-derived bovine immunoglobulins were obtained from edible bovine blood collected at USDA inspected slaughterhouses, therefore is a product that contains a range of immunoglobulins with broad spectrum of antibodies against a variety pathogens and antigens, but doesn’t content specific antibodies developed against particular pathogen or antigen. The experimental diets were formulated to provide similar amount of energy, protein, fat and carbohydrates. They were prepared and irradiated by Harlan Ibérica (Barcelona, Spain), and the composition is shown in Table 1.

**Table 1:** Composition of the experimental diets.

INGREDIENT	Control (g/kg)	SBI diet (g/kg)
SBI†	--	20
Corn starch	199.3	254.8
Skim milk	530.7	430
Sugar	94.5	100
Soybean oil	70	70
Cellulose	50	50
AIN-93 GM‡	35	35
AIN-93 VX‡	15	15
DL-methionine	2.5	2.2
Choline bitartrate	3.0	3.0
Total	1000	1000

^†^SBI: Serum-derived bovine immunoglobulin contains > 90% protein, over 50% of which is IgG. Manufactured according to FDA bulk pharmaceutical ingredient standards by EnteraHealth (Cary, NC) under the commercial name EnteraGam™.

^‡^Provided by Harlan Ibérica (Barcelona, Spain).

### Experimental design

Animals were weaned at 21 d of age and started to consume the experimental diets *ad libitum*. They were maintained in the SPF area until d 28, when they were transferred to a conventional housing area. At 56 d of age, mice were anesthetized by i.p administration of ketamine (90 mg/Kg) and xilacine (10 mg/kg) followed exsanguination by cardiac puncture. The clinical signs of colitis were analyzed using the disease activity index (DAI), which scores weight loss, stool consistency and bleeding, as described elsewhere [15].

### Morphological study

Colon fragments were washed with PBS and fixed overnight with 4% paraformaldehyde, dehydrated in graded ethanol, and embedded in paraffin. Ten μm sections were stained with hematoxylin-eosin according to standard protocols. Samples were observed under blind conditions using an optic Olympus BX41 microscope (Münster, Germany).

### *Lamina propria* cell isolation

Cell isolation was performed as described elsewhere [23]. Briefly, the colon was extracted, washed and everted. The tissue was then incubated in 10 ml of pre-digestion solution (HBSS with 10% FBS, 5 mM EDTA and 1 mM DTT) for 25 min at 37°C in a Thermomixer shaker (Eppendorf, Hamburg, Germany). The cell suspension was eliminated.

The remaining colon was cut, finely minced, and incubated in RPMI 1640 (Invitrogen, Carlsbad, CA) containing 5% FBS, penicillin/streptomycin, 10 nM HEPES, 2 mM L-glutamine and 1500 U/mL collagenase (Invitrogen) at 37°C for 20 min in a Thermomixer shaker.

Cell suspensions were centrifuged at 500*g* for 10 min at 4°C. The pelleted cells were re-suspended in 40% Percoll in complete RPMI. This cell suspension was then carefully layered over 80% Percoll in complete RPMI medium and centrifuged at 1000*g* for 20 min at 20°C. The ring of cells was carefully collected, transferred to a clean tube and centrifuged at 500*g* for 10 min at 4°C. The pelleted cells were re-suspended in PBS-FBS. Cell number and viability were determined using acridine orange and ethidium bromide markers.

### Mesenteric lymph node cell isolation

The leukocytes from MLN were isolated as previously described [12]. Cell number and viability were determined using acridine orange and ethidium bromide markers.

### Cell staining

Sample processing was similar to the procedure described previously [24]. Briefly, staining was performed on 3·10^5^ cells. The surface markers were analyzed with primary antibodies conjugated to fluorochromes. The antibodies used were: anti-CD45R (clone 30-F11), anti-CD3 (145-2C11), anti-CD4 (GK1.5), anti-CD25 (PC61.5), anti-Gr1 (Ly-6G; 1A8), anti-CD68 (FA-11), anti-FoxP3 (FJK-16s) and anti-CD14 (Sa2-8), purchased as fluorescent conjugates from BD Biosciences (Franklin Lakes, NJ) or eBioscience (San Diego, CA). Results were analyzed using the Flowjo software package (Treestar Inc., Ashland, OR).

### Cytokine determination

Samples of colon mucosa were homogenized as previously described [13]. IL-2, IL-6, IL-10, IL-17, MCP-1 and MIP-1β were measured by *Bio-Plex Cytokine Assay*^™^ (Bio-Rad, Hercules, CA) and TGF-β by *TGF*β*1 Emax^®^ ImmunoAssay System* (Promega Corporation, Madison, WI).

### Determination of oxidative stress in colon mucosa

Hydrogen peroxide was measured as an indicator of oxidative stress and it was quantified using the Hydrogen Peroxide Assay Kit (Sigma-Aldrich, St. Louis, MI) according to the manufacturer’s instructions.

### Real-time PCR analysis

RNA extraction and reverse transcription were carried out as described previously [15]. Total RNA was retrotranscribed using an iScript cDNA Synthesis Kit (Bio-Rad). The primers used to detect intercellular adhesion molecule-1 (ICAM-1), catalase and GAPDH were as follows: ICAM-1 forward CAGTCCGCTGTGCTTTGAGAA and reverse GAGGTCTCAGCTCCACACTCT; catalase forward TTTCACTGACGAGATGGCAC and reverse GTGGGTGACCTCAAAGTATCC; and GAPDH forward GGCATTGCTCTCAATGACAA and reverse CCCTGTTGCTGTAGCCGTAT. Real-time PCR was performed on a MiniOpticon Real-Time PCR System (Bio-Rad). Samples were tested in duplicate and the average values were used for quantification, which was carried out by the 2^ΔΔ^CT method [25]. Product fidelity was confirmed by melt-curve analysis.

### Statistical analysis

The data from the experiments are presented as the mean ± SEM. Mean values of normally distributed data were compared using one-way ANOVA followed by a LSM post hoc test. Recruitment of activated neutrophils and oxidative stress were correlated by Pearson coefficient. Statistical tests were performed using SPSS-17.0 software (IBM, Armonk, NY). Differences were considered significant at *p* < 0.05.

## Results

Scores for the disease activity index (DAI), used to quantify the clinical signs of colitis at the end of the experimental period, were three-fold higher in mdr1-/- mice than in WT mice, and SBI administration tended to induce a reduction in DAI scores, as reported elsewhere [15].

### SBI supplementation improves body weight gain in mdr1a-/- mice

**Fig 1:**
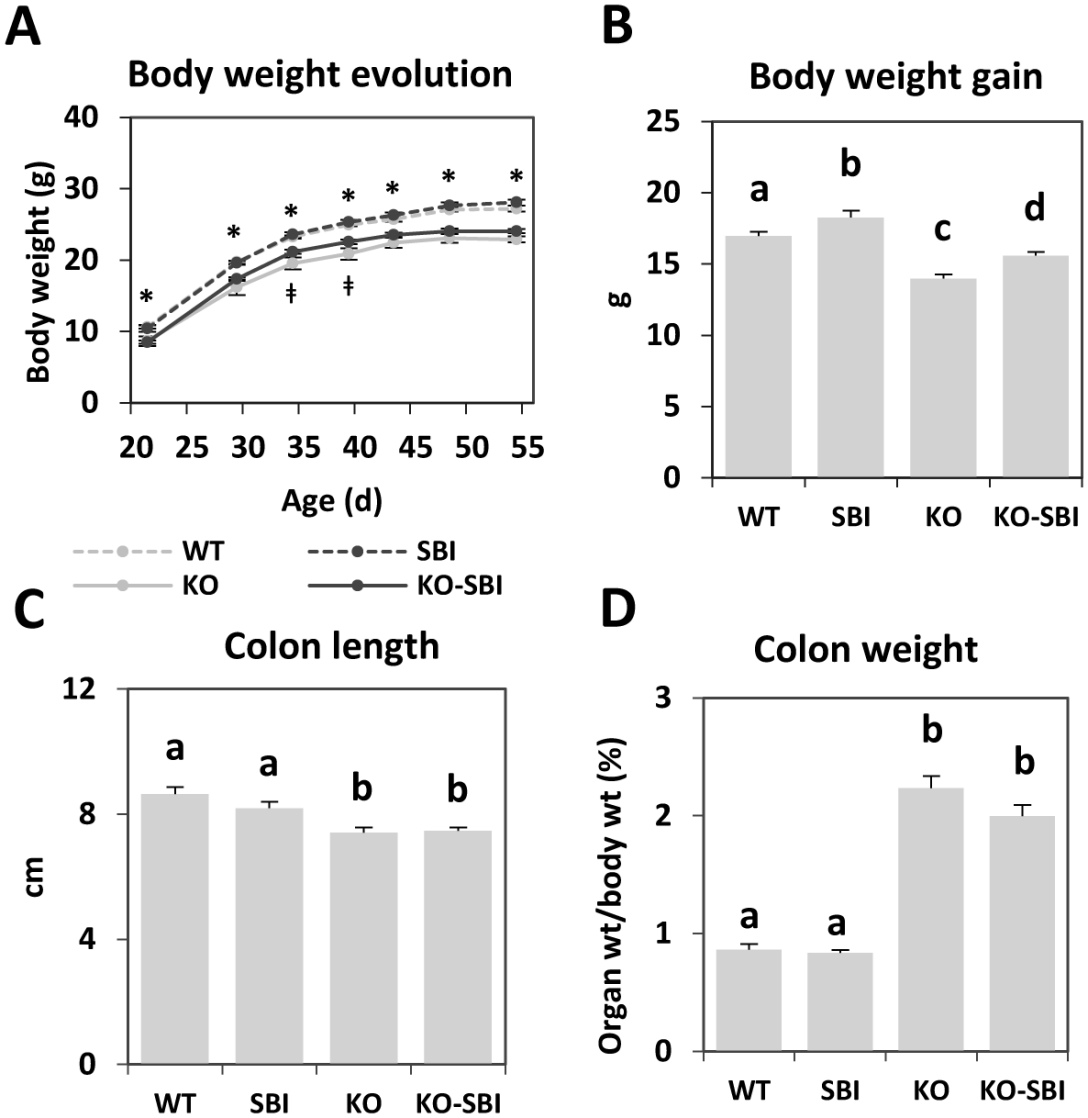
Effects of SBI supplementation on body weight and colon. Panel A shows body weight evolution of WT and KO mice during the experimental period. Results are expressed as mean ± SEM (n = 7–8 animals). *indicates significant differences between WT groups (WT and SBI) and KO groups (KO and KO-SBI); ^**ǂ**^indicates significant differences between KO and KO-SBI groups, P<0.05. Panel B shows body weight gain during the experimental period. Panels C and D show the colon weight and length, respectively. Results are expressed as mean ± SEM (n = 7–8 animals). Means without a common letter differ, *p* < 0.05.

The growth rate of KO mice was lower than that of WT mice, as shown in Fig 1A (*p* < 0.05). Dietary SBI increased body weight throughout the experimental period (comparison of control KO and KO-SBI groups at day 45 and 55 resulted in *p* = 0.07 and *p* = 0.06 values, respectively). At the end of the experimental period, there was a significant effect of SBI supplementation on weight gain in both WT and KO mice (*p* < 0.05; Fig 1B).

### SBI reduces colon infiltration in mdr1a-/- mice

**Fig 2:**
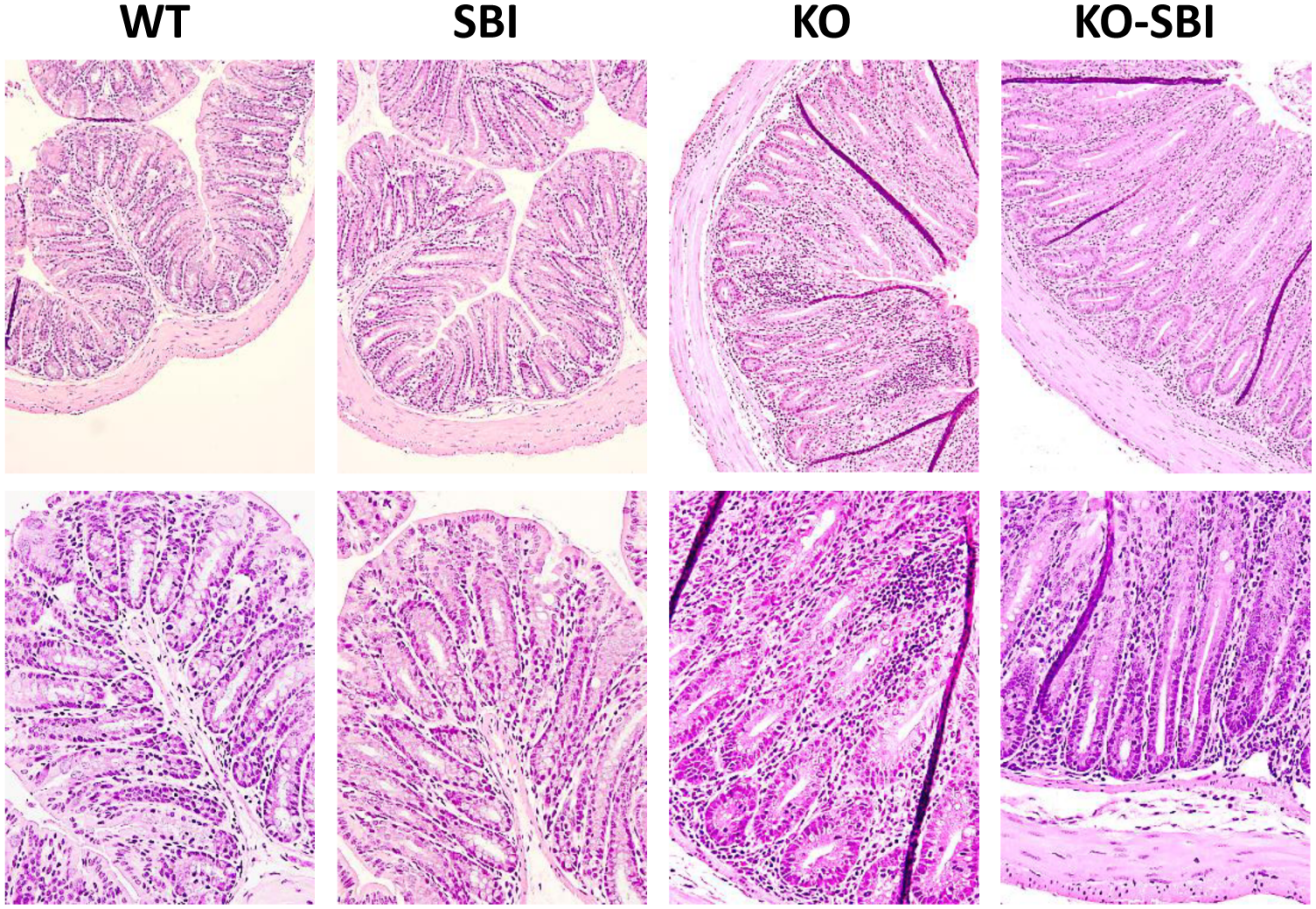
Colon histology. Representative images obtained from the colon of WT, SBI, KO and KO-SBI mice. Upper row, 10x magnification; lower row, 20x magnification.

**Fig 3:**
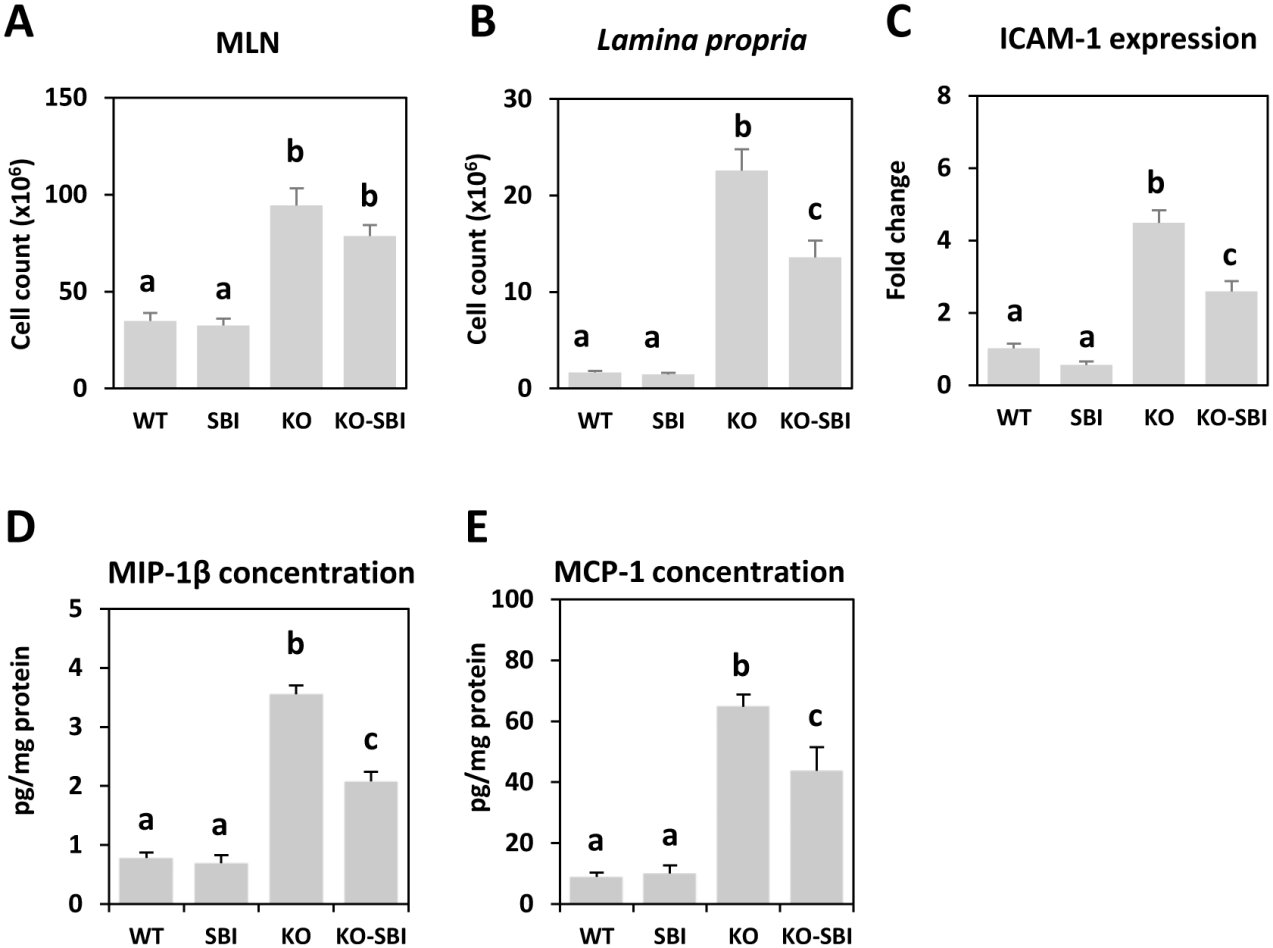
Effects of SBI supplementation on cell recruitment. Panels A and B represent leukocyte counts in mesenteric lymph nodes (MLN) and in the *lamina propria* of the colon, respectively. Panel C shows the expression of intercellular adhesion molecule 1 (ICAM-1) in the colon mucosa. Panels D and E indicate the concentration of MIP-1β and MCP-1 in colon mucosa. Results are expressed as mean ± SEM (n = 7–8 animals). Means without a common letter differ, *p* < 0.05.

The colons of KO mice were thicker and shorter than those of WT animals (*p* < 0.001; Fig 1C and 1D). The differences in colon diameter and wall thickness are clearly evident in the colon sections shown in Fig 2. SBI supplementation reduced colon weight only in KO mice (*p* = 0.06; Fig 1C) and had no effects on colon length. Leukocyte recruitment in mesenteric lymph nodes (MLN) and in *lamina propria* were markedly stimulated in KO mice. There was about 3-fold increase in leukocyte number in MLN, and a 13-fold increase in the *lamina propria* compartment (all *p* < 0.001, Fig 3A and 3B; Fig 2). The interaction between dietary treatment and colitis onset was only apparent in the *lamina propria*, where SBI reduced leukocyte recruitment by 40% (*p* < 0.001).

The expression of markers that participate in leukocyte recruitment, such as ICAM-1, was notably increased in colitic mice (Fig 3C) and reduced in part by dietary SBI (*p* < 0.001). Furthermore, MIP-1β and MCP-1, chemokines involved in leukocyte recruitment into the colon mucosa, were also strongly stimulated in KO mice (both *p* < 0.001; Fig 3D and 3E), and SBI supplementation reduced their concentration in the colon (all *p* < 0.05).

### SBI reduces the activation of innate immunity in mdr1a-/- mice

**Fig 4:**
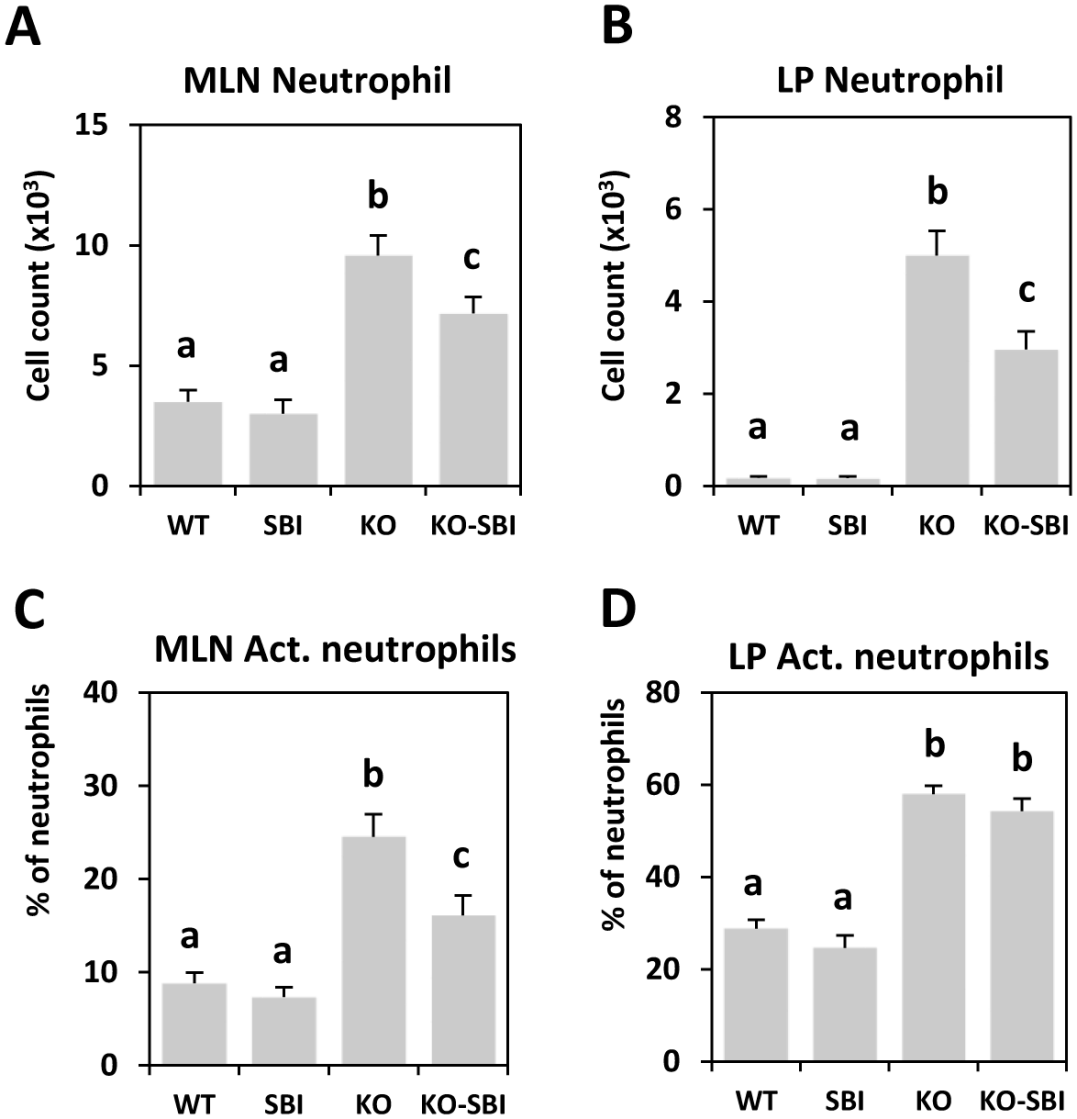
Effects of SBI supplementation on neutrophil recruitment and activation. Panels A and B show neutrophil counts in mesenteric lymph nodes (MLN) and colon *lamina propria* (LP), respectively. Panels C and D show the percentage of activated (Act.) neutrophils with respect to the neutrophil population in MLN and colon LP, respectively. All results are expressed as mean ± SEM (n = 7–8 animals). Means without a common letter differ, *p* < 0.05.

Neutrophil recruitment in KO mice was also enhanced in MLN (2.7-fold; Fig 4A) and in the *lamina propria* (28-fold; Fig 4B), as was the percentage of activated neutrophils (2–3 fold; all *p* < 0.001, Fig 4C and 4D). SBI reduced neutrophil counts and the percentage of activated neutrophils in MLN (both *p* < 0.05); however, in the *lamina propria*, the interaction between colitis onset and SBI supplementation was only observed for neutrophil recruitment (*p* < 0.001; Fig 4B).

**Fig 5:**
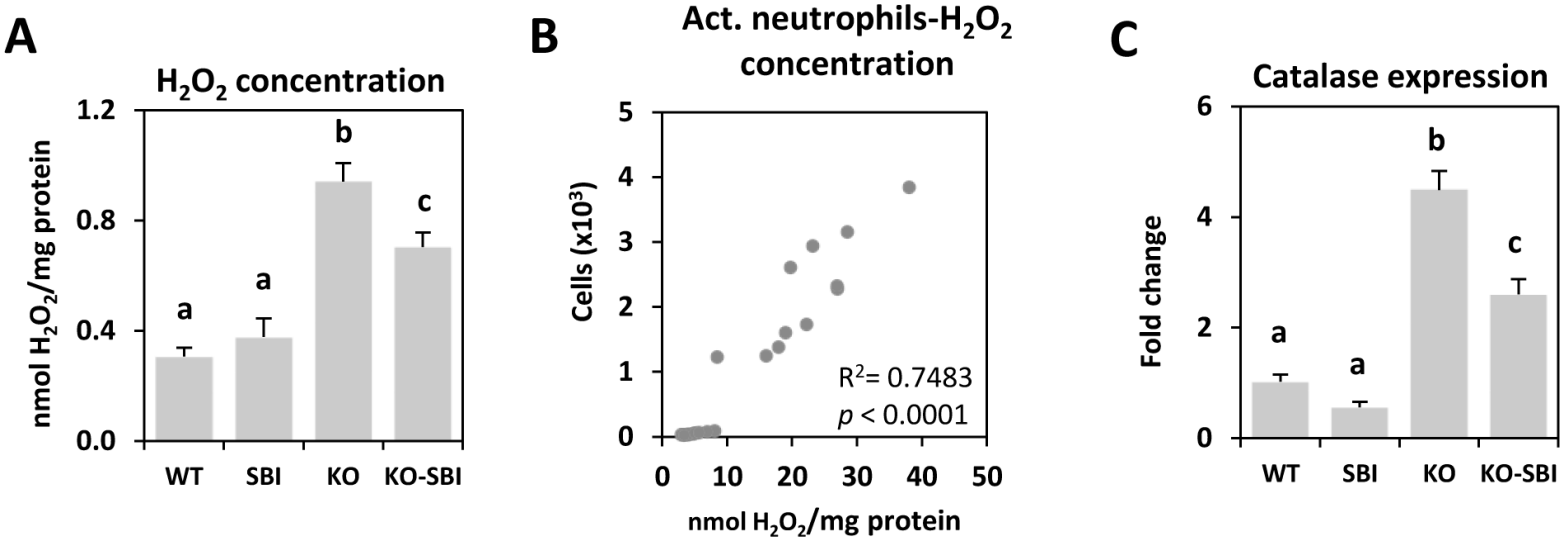
Effects of SBI supplementation on oxidative stress. Panel A shows mucosal oxidative stress measured as hydrogen peroxide concentration in homogenates of colon mucosa. Panel B shows the correlation between infiltrated activated (Act.) neutrophils and oxidative stress. Panel C indicates the expression of catalase in colon mucosa. Results are expressed as mean ± SEM (n = 5–6 mice). Means without a common letter differ, *p* < 0.05.

Another variable used to characterize inflammation was peroxide production and metabolism. KO mice showed an increased concentration of hydrogen peroxide in the colon mucosa and this effect was partially reduced by SBI supplementation (both *p* < 0.05; Fig 5A). The number of activated neutrophils that were infiltrated into the *lamina propria* and peroxide concentration in the colon were well correlated (R^2^ = 0.9215; *p* < 0.0001; Fig 5B), indicating that neutrophils are involved in colitis development. Catalase expression was also strongly stimulated in KO mice and significantly reduced in KO-SBI mice (*p* < 0.05; Fig 5C).

### SBI mitigates the activation of adaptative immunity in mdr1a-/- mice

**Fig 6:**
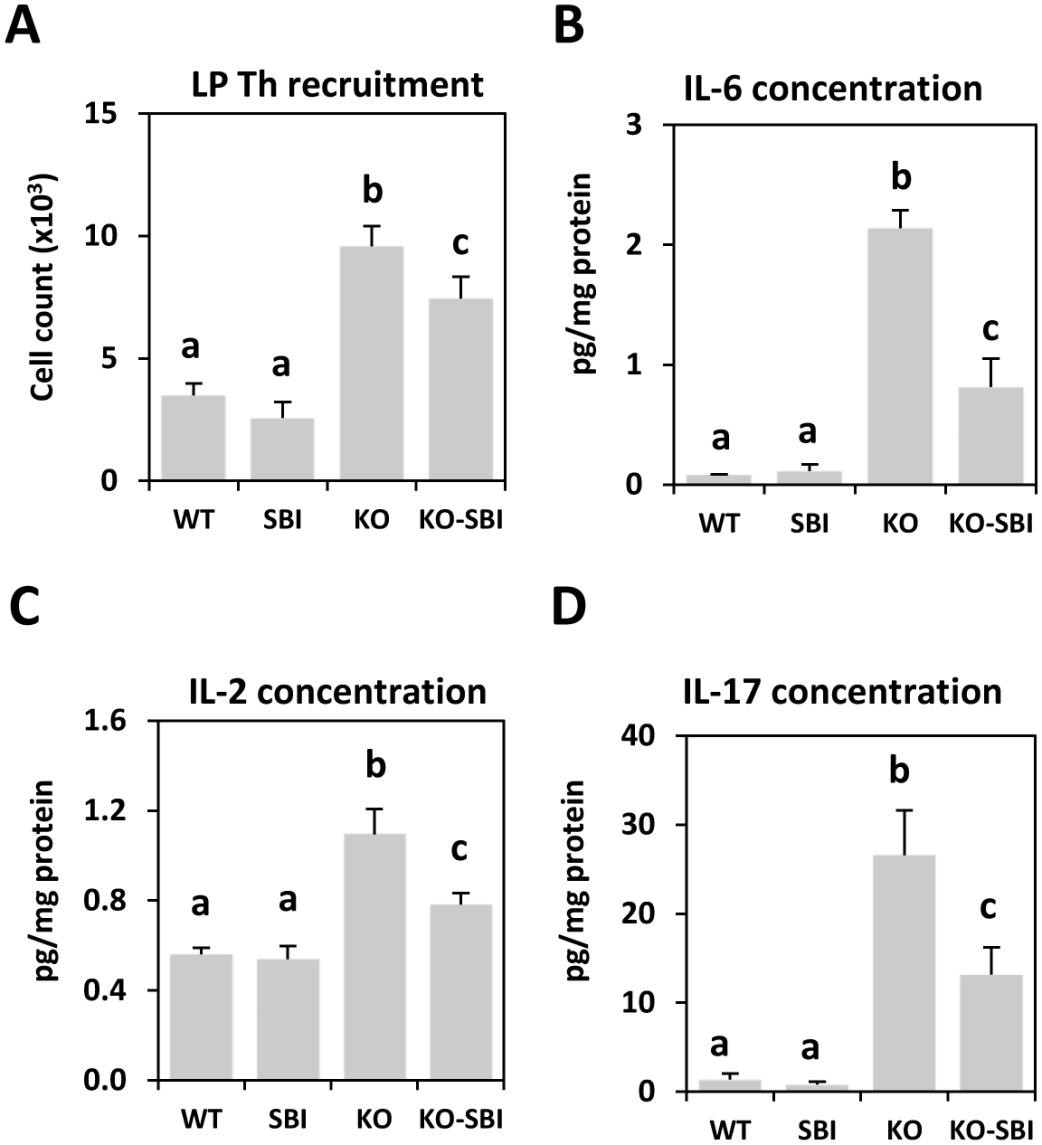
Effects of SBI supplementation on Th lymphocyte recruitment and cytokine production. Panel A shows Th lymphocyte counts in the *lamina propria* (LP) of the colon. Panels B, C and D indicate the concentration of IL-6, IL-2 and IL-17 in colon mucosa, respectively. Results are expressed as mean ± SEM (n = 5–6 mice). Means without a common letter differ, *p* < 0.05.

KO mice showed a considerable increase in Th lymphocyte counts in colon *lamina propria*, and this effect was reduced by SBI (*p* < 0.001 and *p* < 0.05, respectively; Fig 6A).

Pro-inflammatory cytokines were also analyzed in the colon mucosa. As expected, KO mice showed a several-fold increase in IL-6 secretion relative to WT mice (*p* < 0.001, Fig 6B), and this was reduced by SBI (*p* < 0.001). KO mice also had an increased IL-2 production (*p* < 0.001; Fig 6C), which was attenuated by dietary supplementation (*p* < 0.005). In addition, the release of IL-17, a cytokine that mediates recruitment of monocytes and neutrophils to the site of inflammation [26], was strongly stimulated in the colonic mucosa (*p* < 0.001; Fig 6D). The increased production of IL-17 observed in colitic mice was reduced by SBI (*p* < 0.005). In contrast, the production of IL-4, a cytokine typically involved in the Th2 immune response, was not affected by the onset and progression of colitis, and no effect of SBI was observed either (data not shown).

**Fig 7:**
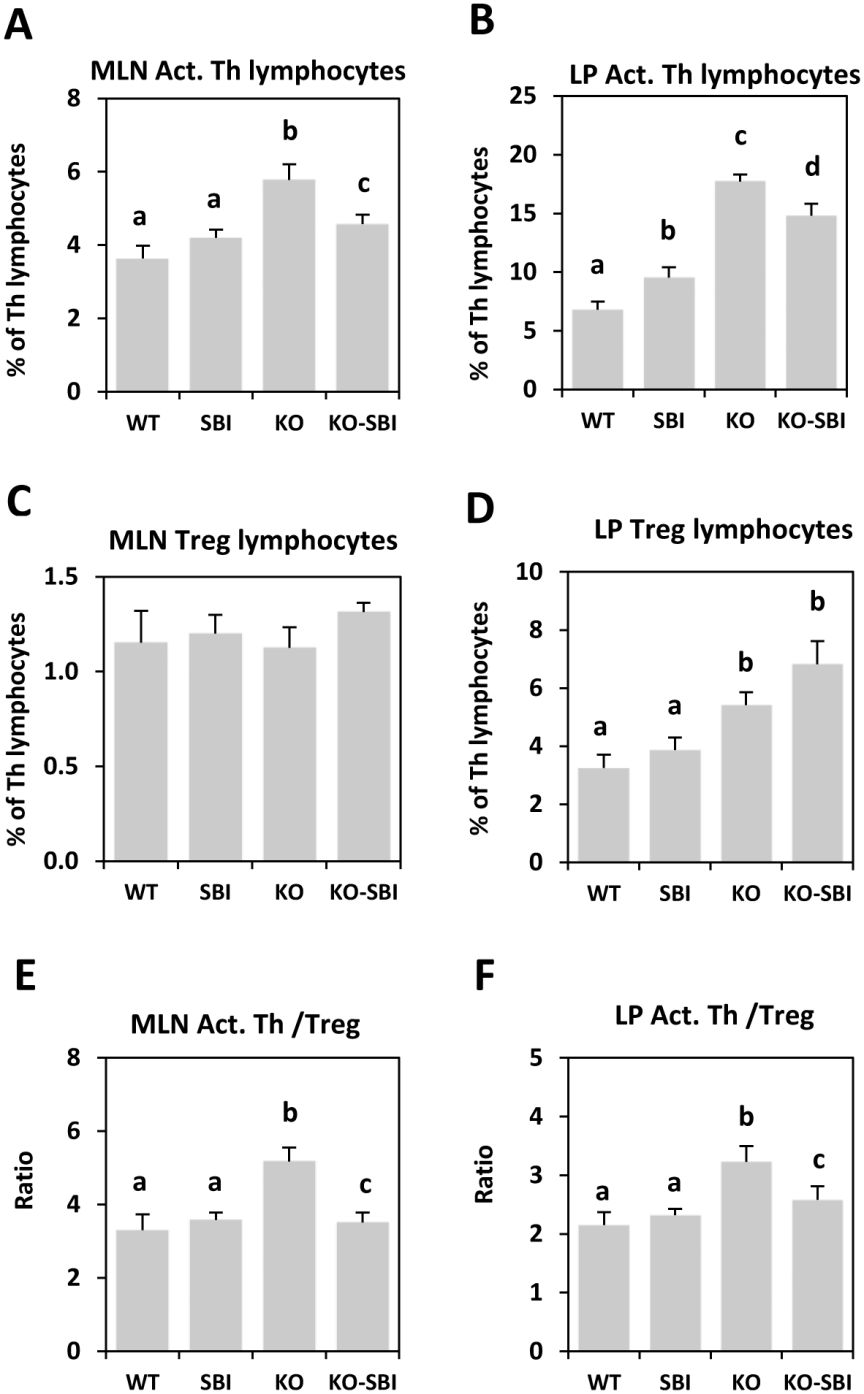
Effects of SBI supplementation on activated (Act.) and regulatory Th lymphocytes (Treg). Panels A and B show the percentage of Act. Th lymphocytes in mesenteric lymph nodes (MLN) and in the *lamina propria* (LP) of the colon. Panels C and D show the percentage of Treg in MLN and LP. The ratios between both populations are shown in panels E and F. Results are expressed as mean ± SEM (n = 5–6 mice). Means without a common letter differ, *p* < 0.05.

The percentage of activated lymphocytes in MLN and *lamina propria* of KO mice was increased, and this change was partially prevented by SBI supplementation (*p* < 0.001 and *p* < 0.05 respectively; Fig 7A and 7B). The Treg population was increased in the *lamina propria* of KO mice (*p* < 0.01; Fig 7D) and this was not affected by SBI supplementation. Calculation of the ratio of activated to regulatory Th lymphocytes (the Tact/Treg ratio) showed that this variable was markedly increased in MLN and *lamina propria* during the development of colitis (all *p* < 0.005, Fig 7E and 7F), as a result of an increased proportion of Tact lymphocytes. This change was reverted by dietary SBI (*p* < 0.05), indicating lower immune activation.

**Fig 8:**
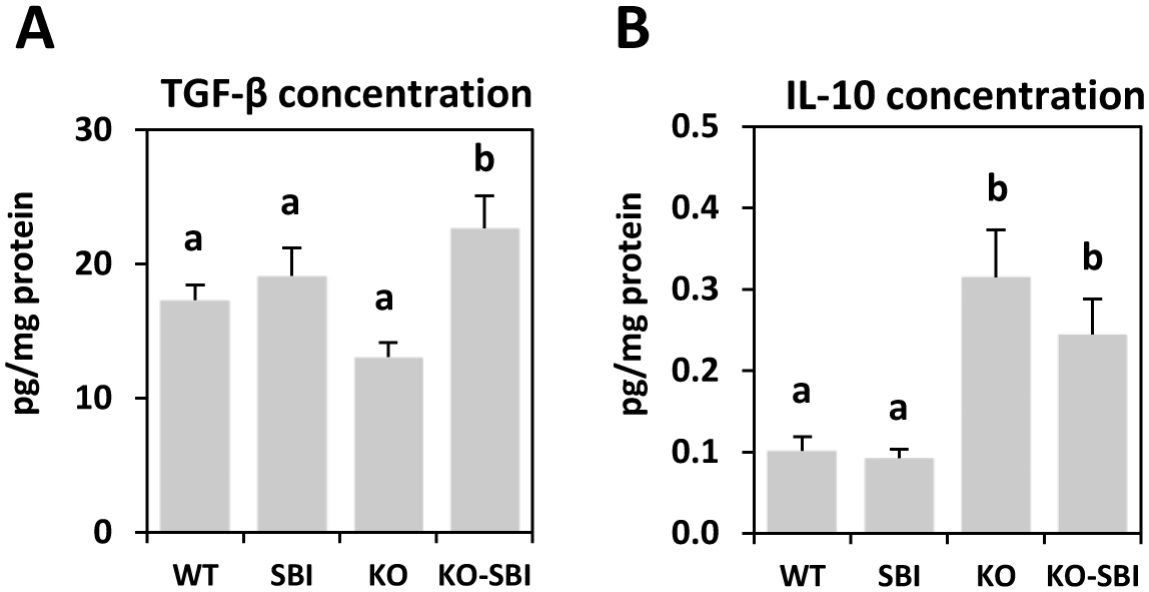
Effects of SBI supplementation on TGF-β (A) and IL-10 (B) production in the colon. Results are expressed as mean ± SEM (n = 5–6 mice). Means without a common letter differ, *p* < 0.05.

Lastly, the production of mucosal anti-inflammatory cytokines was also studied. Distinct effects on IL-10 and TGF-β were observed. The progress of colitis did not affect TGF-β secretion (Fig 8A), but IL-10 production was markedly increased in KO mice (*p* < 0.001; Fig 8B). The effects of dietary supplementation with SBI also differed, observing TGF-β production (*p* < 0.005) without affecting the IL-10 balance.

## Discussion

Mdr1a-/- mice are prone to spontaneously develop severe intestinal inflammation, with clinical and histopathological manifestations that are phenotypically similar to human IBD [27;8]. Recently, alterations in colon barrier function and increased expression of the pro-inflammatory cytokines TNF-α and IFN-γ have been reported in mdr1a KO mice [15], and it has also been shown that dietary supplementation with SBI reduces the expression of pro-inflammatory markers and prevents barrier deterioration. In the present study, we further characterize mucosal GALT during progression of the colitis syndrome, and describe how these changes can be modulated by dietary intervention.

A hallmark of all chronic inflammatory disorders is the rapid recruitment and inappropriate retention of leukocytes at the site of inflammation [28]. The KO mice used in the present study showed increased leukocyte recruitment into *lamina propria* and MLN, which correlated well with increased expression of TNF-α [15] and of MIP-1β and MCP-1. They also showed increased ICAM-1 expression, an adhesion molecule that plays an important role in evoking effective immune responses [29] and which is critical for T cell activation and leukocyte recruitment to the site of inflammation.

Leukocyte recruitment into the *lamina propria* was attenuated by SBI. This effect is mediated by two pathways working in parallel: firstly, SBI reduces expression of TNF-α [15], which is the main regulator of ICAM-1 expression. This enables activated leukocytes to bind endothelial cells via the ICAM-1/integrin signaling pathway, which can then transmigrate into tissues [30]. Secondly, SBI reduces the expression of MIP-1β and MCP-1, two chemokines involved in leukocyte recruitment [31] that have been shown to be increased in the intestinal mucosa of patients with CD [32]. Overexpression of MCP-1 has been reported to induce fibrogenic responses in mouse colon, due to interactions between T cells and fibroblasts/myofibroblasts [33].

SBI supplementation reduced the accumulation of activated innate immune cells in both MLN and *lamina propria*, and this was correlated with reduced production of MIP-1β and MCP-1. This effect is noteworthy because chemokines are important mediators for the recruitment and accumulation of neutrophils and macrophages in colitis models [34;35]. Moreover, the effects on cellular infiltration are important because activated neutrophils and monocytes produce reactive oxygen species (ROS) and free radicals that increase oxidative stress, which may further injure the tissue, as enhanced ROS formation disrupts epithelial cell integrity [36]. SBI supplementation reduced oxidative stress in the colon mucosa and improved the epithelial barrier function, as already observed elsewhere [15]. The effect of SBI on ROS production is probably the result of reducing the recruitment of activated innate cells rather than a consequence of increased colon antioxidant capacity. SBI did not affect the expression of antioxidant enzymes, such as catalase, but it reduced the number of activated neutrophils in the *lamina propria* of the colon, which in turn will reduce the expression of iNOS in the colon, as previously shown [15].

Studies using models of IBD have shown that CD4+T cells play a major role in initiating and shaping the syndrome and that their capacity to extend gut inflammation is largely dependent on the production of distinct cytokine profiles [37]. In the mdr1a KO mouse model, the percentage of activated Th lymphocytes in MLN and in *lamina propria* was increased, and this correlated well with the expression of IFN-γ [15] and IL-2 and IL-17 secretion, as described for IBD [38]. Increased activation of *lamina propria* T-lymphocytes is a key factor in IBD pathogenesis, as it leads to unrestrained accumulation of activated lymphocytes that perpetuate inflammation [39;40;27]. However, production of mucosal IL-4 (a cytokine typically representative of ulcerative colitis syndrome) was not affected in mdr1 KO mice (data not shown), indicating that the mdr1 KO model may be more representative of the CD syndrome [21].

SBI supplementation reduced the percentage of activated Th lymphocytes in MLN and in the *lamina propria*, and this resulted in lower IL-2 and IFN-γ release. This is noteworthy because there is broad consensus that Th lymphocytes are the most important activated immune cells involved in IBD pathogenesis [41]. Pathological T cell activation results in a disturbed balance between pro- and anti-inflammatory cytokines [42]. Moreover, SBI supplementation reduced IL-17 secretion in colon mucosa of mdr1a KO mice. This effect may be clinically relevant because the inflammation profile that has been described for CD is characterized by IL-17 activation that further stimulates the secretion of pro-inflammatory cytokines in a self-sustaining cycle [43]. SBI stimulated the production of mucosal TGF-β and this was inversely correlated with IL-17 production, indicating that SBI-dependent changes in cellular and cytokine pro-inflammatory profiles in mdr1a KO mice are mediated by TGF-β rather than by IL-10. Taken together, the present results indicate that CD4+T cells play an important role in shaping the immune response during development of colitis and that attenuation of Th1 and Th17 responses by SBI could represent a significant step forward in the treatment of IBD. This is especially so in the case of CD, given that increased expression of Th1 and Th17 in this syndrome is well documented [2].

The homeostatic condition of the intestinal mucosa is a state of *controlled inflammation* characterized by a balance between protective immunity and tolerance to self-antigen and commensal bacteria [44]. Tolerance is maintained by Tregs, a population of CD4+ T cells that controls immune responses by inhibiting the proliferative and effector functions of other T cells. There is evidence showing that the inflammation that characterizes IBD may be caused by the loss of homeostatic steady-state between Treg (CD4+ CD25high Foxp3+) and pro-inflammatory activated Th cells [45]. Mdr1a KO mice have an increased Tact/Treg ratio in MLN and *lamina propria* due to an increase in the Tact population, and SBI partially prevents this change because it reduces the Tact subpopulation and increases the percentage of Treg lymphocytes. The important result is that SBI can reduce the Tact/Treg lymphocyte ratio in both tissues, supporting a bias toward immune tolerance. This effect of SBI is homeostatic, as it restores the balance between Treg and Tact lymphocytes that was altered in colitic mice. A similar pattern of response has been observed in mice models of toxin-induced acute mild intestinal inflammation [46] and acute lung inflammation [47]. In both cases, the toxin challenge increased the Tact/Treg ratio and dietary plasma proteins restored the pre-challenge Tact/Treg ratio values. The importance of these effects lies in the role of Tregs in the suppression of Th effector cells through the secretion of anti-inflammatory cytokines such as IL-10 and TGF-β [48]. IL-10 reduces Th cell proliferation and decreases secretion of harmful cytokines, and TGF-β has similar effects on naïve and already differentiated Th cells [49]. Thus, the suppressant effect of Treg on Tact cells is important for immune homeostasis in the gastrointestinal tract. SBI supplementation did not modify the release of IL-10, but it did increase TGF-β production, suggesting that the effects of SBI on cell activation are mediated by changes in TGF-β production.

In summary, dietary SBI reduced the amount of activated Th lymphocytes and promoted the presence of regulatory Th cells in mice spontaneously developing colitis; in turn, this increased TGF-β secretion favored the predominance of an anti-inflammatory immune profile. This cascade of effects eventually reduces mucosal pro-inflammatory cytokines. The beneficial effect of SBI on this model suggests a role of serum-derived supplements in the prevention and amelioration of IBD.
